# Device-based therapies in cardio-renal syndrome: a pathophysiology-driven approach to a complex bidirectional disease

**DOI:** 10.3389/fcvm.2026.1795540

**Published:** 2026-04-02

**Authors:** Gift Echefu, Ryan Sullivan, Ifeoluwa Stowe, Paschalyn Ukwuani, Adedayo Adeboye, Damodar Kumbala

**Affiliations:** 1 Department of Cardiovascular Medicine, University of Tennessee Health Science Center, Memphis, TN, United States; 2 Department of Nephrology, Beth Israel Deaconess Medical Center/Harvard Medical School, Boston, MA, United States; 3 University of the Visayas College of Medicine, Cebu, Philippines; 4 Department of Nephrology, Renal Associates of Baton Rouge, Baton Rouge, LA, United States

**Keywords:** venous congestion, renin–angiotensin–aldosterone system, cardiorenal syndrome, device-based therapies, diuretic resistance in heart failure, DRI2P2S classification, microcirculation–interstitium–lymphatic axis, heart failure therapeutics

## Abstract

Cardiorenal syndrome (CRS) is a complex clinical condition characterized by the simultaneous relationship between cardiac and renal dysfunction, often complicating the management of heart failure. Despite advancements in guideline-directed medical therapy, persistent congestion and diuretic resistance continue to be prevalent and are closely linked to negative outcomes. Device-based therapies have shown potential as adjunctive strategies to address critical pathophysiologic mechanisms of CRS not adequately addressed by pharmacologic approaches alone. This review offers a current perspective on device therapies for CRS, presented in accordance with their predominant mechanistic effects on volume regulation and hemodynamics. It is crucial to underscore the necessity of individualized patient selection, which is determined by the underlying hemodynamic phenotype, renal sodium avidity, diuretic responsiveness, and congestion burden, rather than isolated changes in renal function. We suggest a phenotype-driven stepwise management implementation algorithm to potentially aid appropriate timing and selection of device-based interventions. Also considered are practical considerations including procedural risk, integration into heart failure care pathways, and the role of device therapy in a broader disease-modifying strategy.

## 1 Introduction

Heart failure (HF) is prevalent, with around 6.7 million patients over 20 years old diagnosed in the US, a figure projected to rise to 8.7 million by 2030 (1). Additionally, HF-related mortality continues to increase annually, with a concordant alarming increase in mortality related to CRS (2). Admission for acute decompensated heart failure (ADHF) is also the leading cause of hospitalization in the US and Europe, and post-hospital discharge and re-admission rates are worsening (3).

The complex pathophysiologic relationship between the heart and the kidneys has been well described in the literature. Although the classification of CRS has changed over the years, the current classification endorsed by the American Heart Association sorts this complex group of diseases into 5 main subtypes (4). The earlier description of CRS as primarily a state of hemodynamic impairment culminating in renal dysfunction has been replaced by current understanding that CRS results from interplay between hemodynamic and non-hemodynamic factors. This bidirectional cardiac and renal signaling creates an increasing feedback loop that promotes progressive renal and cardiac dysfunction. In CRS, hemodynamic variables, particularly renal hypoperfusion and venous congestion, are primary contributors; while non-hemodynamic processes such as RAAS activation, sympathetic nervous system stimulation, and oxidative stress all significantly maintain cardio-renal injury (5).

Approaching CRS as a systemic syndrome rather than isolated organ dysfunction underscores the prognostic significance of alterations in traditional markers like serum creatinine or eGFR in acute heart failure contexts, as they suggest ongoing pathophysiological interactions that adversely affect clinical outcomes (6). Patients admitted to the hospital for acute decompensated heart failure (ADHF) and cardiorenal syndrome (CRS) often present management challenges especially when renal biomarkers continue to rise despite aggressive diuresis. Because of this, most patients admitted to the hospital with ADHF and CRS fail to achieve an euvolemic state prior to discharge, and this contributes to repeated hospitalizations. This could be, in part, related to diuretic resistance which remains a poorly understood aspect of the care of HF patients. “Diuretic braking” is a term that refers to an observed phenomenon in which patients have a reduced urinary response to repeated doses of intravenous loop diuretics, and this phenomenon has been described previously (7). Given this clinical observation, diuresis alone may not be sufficient for the management of certain individuals with ADHF and CRS, creating a potential role for device-based therapies in these patients. The current landscape of device-based therapies for the management of CRS can be simplified by categorizing them based on the mechanisms by which they improve hemodynamics and modulate renal perfusion. Rosenblum et al. grouped these devices using the DRI2P2S classification system, dilators, removers, inotropes, interstitial, pushers, pullers, and selective. In contrast to pharmacological therapies for CRS which often have secondary mechanisms of action, device-based therapies more specifically target deleterious mechanisms of decompensation and diuretic resistance in heart failure (8). This contemporary review focuses on the emerging devices currently being applied or under investigation for the management of ADHF, with implications for use in CRS, and their effects on urine output and renal biomarkers. Specifically, we focus on pushers, pullers, and fluid shifter devices and their relevance to the management of CRS.

## 2 Pathophysiological framework of CRS

Clinically, CRS is classified into five subtypes. Type 1 CRS describes acute cardiac dysfunction leading to acute kidney injury, while Type 2 involves chronic heart failure resulting in progressive chronic kidney disease. Type 3 CRS characterizes acute renal dysfunction precipitating acute heart failure, and Type 4 encompasses chronic kidney disease contributing to chronic heart failure. Type 5 CRS arises from systemic disorders, including sepsis or inflammatory diseases (such as sarcoidosis) and liver cirrhosis, resulting in concurrent cardiac and renal dysfunction. The limitations of this scheme stem from its dependence on the identification of a distinct “primary” organ insult and the determination of chronicity or acuity based on clinical data, often unavailable or ambiguous (9). In real patients exhibiting concurrent cardiac and renal dysfunction, accurately assigning a category may be challenging, therefore limiting its clinical application and therapeutic guidance (9).

The DRI2P2S classification offers the clincal advantage of categorizing these patients based on the pathophysiological drivers and therapeutic targets in CRS (Table 1). In this classification, CRS is regarded as a complex bidirectional interaction between cardiac and renal dysfunction driven by intertwined hemodynamic, neurohormonal, inflammatory, and microvascular mechanisms. The Acute Dialysis Quality Initiative categorization of Cardiorenal Syndrome addressed the early ambiguities in CRS definition and assisted clinicians in administering phenotype-based, goal-directed therapy for Cardiorenal Syndrome at the point of care. Central to CRS pathophysiology is venous congestion, which has emerged as a dominant determinant of worsening renal function. Elevated central venous pressure leads to increased renal venous pressure, reduced trans-glomerular filtration gradient, interstitial edema, impaired renal perfusion, and progressive decline in glomerular filtration rate. Additionally, diminished forward cardiac output limits renal arterial blood flow, exacerbating renal ischemia despite maintained systemic blood pressure. Under normal physiologic conditions, increase in cardiac atrial pressure from elevated blood volume or pressure initiates the atrial-renal reflex (also known as the Bainbridge reflex) via the vagus nerve to the kidneys, leading to a reduction in sympathetic nerve activity, reduce renin release promoting natriuresis and diuresis. In CRS, this mechanism becomes impaired resulting in a persistent activation of the sympathetic efferent pathway (10). Neurohormonal activation further amplifies this process as reduced effective circulating volume triggers activation of the renin–angiotensin–aldosterone system and sympathetic nervous system, promoting sodium retention, vasoconstriction, and maladaptive myocardial and renal remodeling (10). These pathways contribute to diuretic resistance and perpetuate volume overload despite escalating pharmacologic therapy.

**Table 1:** Comparative overview of device characteristics and clinical trial parameters.

Category (DRI₂P₂S)	Representative device/therapy	Mechanism of action	Study size/setting	Key endpoints reported	Trial phase/status
Dilators (D)	*Splanchnic nerve modulation*	Increases venous capacitance → reduces central congestion	Small feasibility studies	Hemodynamic decongestion, CVP reduction	Early/preclinical to pilot
Removers (R)	– *Reprieve System*	Direct removal of sodium and fluid	Reprieve system trials	↑ Urine output, ↓ CVP/creatinine	Early clinical experience
			– TARGET-1		
			– TARGET-2		
	– *Alfapump*	Direct removal of sodium and fluid	Alfapump trials	↑ Urine output, ↓ CVP/creatinine	Early clinical experience
			– RED DESERT		
			– SAHARA		
Interstitial (I₂)	– *WhiteSwell*	Enhances lymphatic/interstitial fluid removal	WhiteSwell eLym	Interstitial decongestion	Early clinical evaluation
			– DELTA-HF, multicenter, single arm ∼ plans to recruit 50 patients		
	– *AquaPASS*	Enhances lymphatic/interstitial fluid removal	Small proof of concept case series ∼ 16 HF patients	Interstitial decongestion	Early clinical evaluation
	– *RenalGuard*	Enhances lymphatic/interstitial fluid removal	Case series ∼ 19 patients	Interstitial decongestion	Early clinical evaluation
Pushers (P₁)	– *Aortix percutaneous pump*	Increases renal arterial perfusion & reduces afterload	– Aortix CRS study	↑ urine output, improved perfusion	Ongoing studies
			– DRAIN-HF ∼ plans to recruit 268 patients		
	*Reitan catheter pump*	↑ Renal perfusion via descending aortic support	Multicenter study ∼ 20 patients	Urine output, CI, creatinine change	Early trials
	*Second Heart Assist*	Aortic pump to augment flow	– Feasibility case series ∼ 20	Hemodynamic improvement (cardiac index, LV unloading)	Preclinical/pilot
Pullers (P₂)	*Doraya renal flow regulator*	↓ renal venous/central venous pressures	– DORAYA trial ∼ 9 patients	↑ urine output	Early feasibility
	*preCARDIA system*	Reduces cardiac preload via SVC occlusion	VENUS_HF early feasibility study, multicenter, prospective ∼ 30 patients	↓ venous pressure	Pilot clinical
	*Magenta TRVD (renal venous decongestion)*	IVC venous modulation	Feasibility study ∼ 13 patients	CVP/BNP, renal function	Preclinical
			– Clinical trial terminated		
	*JuxtaFlow Renal Assist Device (RAD)*	Intra-renal pressure modulation	VOID-HF trial, mulitcenter, single arm ∼ 7 patients	Increase in 24 h urine output and sodium excretion	Feasibility
	*Nephronyx perfuser system*	Enhance renal venous outflow and reduce venous congestion	– Small single arm clinical trial	Renal hemodynamics	Feasibility
Selective (S)	*Benephit catheter (Selective delivery)*	Intrarenal vasodilator drug infusion	Early pilot	Hemodynamics	Pre-clinical/feasibility

Inflammatory and fibrotic pathways play a critical downstream role in CRS. These result from sustained venous congestion inducing renal hypoxia, oxidative stress, endothelial dysfunction, and the activation of profibrotic signaling cascades. These processes lead to irreversible structural changes within both the myocardium and renal parenchyma, reinforcing cardiorenal disease progression. Dysfunction of the microcirculation interstitium lymphatic (MIL) axis is another, increasingly recognized, contributing factor to CRS. Under normal physiological conditions, fluid transits from the intravascular compartment into the interstitium and is subsequently cleared by the lymphatic system. In CRS, elevated venous pressures impede lymphatic drainage while increasing capillary filtration, resulting in interstitial edema and tissue hypoxia. Disruption of this axis contributes to peripheral edema, pulmonary congestion, and impaired organ oxygenation, providing a mechanistic rationale for device-based therapies that target interstitial fluid and lymphatic flow.

### 2.1 Diuretic resistance and sodium avidity in CRS

Under normal conditions the kidneys regulate maintain sodium equilibrium via integrated tubular sensing, hemodynamic regulation, and neurohormonal modulation to preserve extracellular volume and blood pressure. The macula densa in the distal tubule serves as a primary sensor, detecting sodium chloride (NaCl) supply through the NKCC2 transporter. Elevated NaCl levels inhibits renin secretion, diminishing the activation of the RAAS and facilitating natriuresis (11). These mechanisms enable the kidneys to regulate sodium excretion in accordance with intake, thereby preserving circulatory homeostasis. Impairment in sodium sensing or signaling can lead to inappropriate sodium retention, even in the presence of volume overload. In acute heart failure, decreased effective arterial blood volume, venous congestion, and impaired renal perfusion affect chloride delivery to the macula densa triggering renin release regardless of actual intravascular volume status, resulting in inappropriate activation of the RAAS. The neurohormonal response facilitates efferent arteriolar constriction, sodium and water retention, and renal vasoconstriction, mechanisms directly leading to the deterioration in renal function and sustaining CRS (12). This perspective provides mechanistic insight into why CRS is often resistant to standard volume-based therapies and why renal dysfunction can persist despite apparent decongestion. In acute heart failure patients presenting with renal dysfunction lower urinary sodium has been associated with risk of longer hospitalization (13), severe cardio-renal disease (14), poor 90-day HF hospital free survival and all-cause mortality (15).

Diuretic resistance reflects a state of heightened renal sodium avidity driven by impaired renal perfusion, tubular adaptation, neurohormonal overactivity, hemodynamic stress, and chloride-mediated transport regulation. Loop diuretics remain first-line for decongestion in acute and chronic heart failure, yet up to 20%–30% of patients exhibit inadequate natriuresis and persistent congestion despite aggressive dosing, defining clinical resistance (16). Patients presenting with acute heart failure may respond to diuretics initially, however, as natriuretic efficiency diminishes, there is often a need for increased dosages or the addition of another class of diuretic to achieve sequential nephron blockade (16). The impaired response results from decreased renal perfusion, venous congestion, and reduced tubular delivery of diuretics, along with ongoing activation of sodium-retentive neurohormonal pathways such as the renin–angiotensin–aldosterone system and the sympathetic nervous system (10).

Diuretic resistance encompasses more than medication inefficacy, indicating a self-perpetuating sodium-retentive renal state. Reduced cardiac output and increased venous pressures in turn reduce renal perfusion and restrict tubular delivery of diuretics, thus decreasing drug availability at the sites of action (16). Chronic exposure to diuretics activates adaptive mechanisms, including distal nephron remodeling and increased expression of sodium transporters, which improve sodium reabsorption downstream and reduce overall natriuresis (16). In CRS, these adaptations are exacerbated by hemodynamic compromise, resulting in the kidney adopting a sodium-conserving phenotype despite the implementation of aggressive diuretic therapy.

## 3 Classification of device based therapies: the DRI2P2S framework

### 3.1 Dilators

Dilator devices aim to reduce systemic vascular resistance and redistribute blood volume away from the central circulation. One example is splanchnic nerve blockade (SNB), which targets the large venous capacitance of the splanchnic circulation. By decreasing sympathetic tone in the vascular bed, these devices reduce preload and central venous congestion without directly altering cardiac contractility.

In SNB, an anesthetic, either lidocaine or ropivacaine is percutaneously injected into the splanchnic nerve. The block induces acute sympathetic denervation of the splanchnic vascular bed, which results in increased venous capacitance, decreased stressed blood volume, and lowered central filling pressures. Hemodynamics and symptoms are monitored for 60–90 min, indicating the temporary nature of anesthetic effects (4–16). Early clinical studies suggest potential benefits in reducing filling pressures and improving symptoms, although long-term renal outcomes remain under investigation. Current evidence is from non-randomized, open label, proof of concept investigations (17–19). Clinical utility and application are limited by several factors including study designs, small cohort sizes, lack of renal endpoints, brief intervention duration, and indirect mechanistic evidence. Additionally, most evidence pertains to decompensated HF with reduced EF rather than cardiorenal syndrome which often includes HFpEF and primary renal dysfunction (17). Mechanistic relevance may differ across these syndromes given distinct neurohormonal and hemodynamic characteristics. Further rigorous, controlled, long-term trials, especially those incorporating comprehensive renal assessments, are necessary before splanchnic nerve modulation can be reliably integrated into therapeutic strategies for cardiorenal syndrome.

### 3.2 Removers

As noted, congestion is a critical contributor to cardiorenal syndrome. Managing congestion is associated to enhanced clinical outcomes and a decrease in hospitalization rates and may also avert the progression of kidney disease related to renal venous congestion (20). Ultra filtration (UF) through acquapheresis and peritoneal dialysis, facilitates the mechanical removal of fluids and electrolytes without modifying the plasma ion concentration, yielding isotonic and iso-osmolar ultrafiltrate producing more isotonic urine (21, 22). It inhibits the activation of the renin-angiotensin-aldosterone system and lowers the hydrostatic pressure in the nephrons (21, 22). The evidence reporting on the outcomes and efficacy of UF is variable with many supporting its role in improving renal function (23–26). The inconsistent results may be related to the UF protocols, prevailing patient hemodynamics and clinical condition. UF rates requires adjustment commensurate to patient's vital signs as higher rates could lead to reduced preload and hypotension especially among patient with right heart failure and heart failure with preserved ejection fraction (27, 28).

#### 3.2.1 Alfapump (direct sodium removal)

The Aflafpump is a surgically implanted device that directly extracts sodium-rich fluid from the peritoneal cavity via tunneled catheters, enabling continuous low-volume drainage that is eliminated by the bladder. Peritoneal dialysis has been used to manage cases of refractory heart failure, with small studies indicating improvements in symptoms, quality of life, and neurohormonal activation among patients experiencing refractory congestion, even when treated with high-dose loop diuretics (29). The concept of direct sodium removal (DSR) involves the use of the peritoneum to remove large amounts of sodium using sodium-free peritoneal solutions. In 2020, the first-in-human experience of DSR was studied in 10 patients undergoing peritoneal dialysis (PD) (30). Superior sodium removal was achieved in the DSR group compared to the traditional PD group. Additionally, the procedure was noted to be well-tolerated and had a limited effect on serum potassium levels and other electrolytes.

The RED DESERT (NCT04116034) and SAHARA (NCT04882358) direct sodium revmoval device trial results are preliminary, single-arm feasibility studies investigating implanted alfapump system. In RED DESERT, small patient cohorts demonstrated that DSR was feasible and generally safe, significantly enhancing diuretic response (increased sodium excretion), facilitating continued lowering or termination of high-dose loop diuretics, and correlating with improved kidney function and decreased heart-failure biomarkers over a follow-up period of approximately one year (31). Similarly, Interim results from SAHARA demonstrate that DSR effectively reduces congestion and normalizes diuretic response, accompanied by weight loss and improved NT-proBNP levels, without significantly impairing renal function (31). Both studies are limited by their small size, non-randomized, single-arm proof-of-concept designs, which restrict causal inference and generalizability. Further large-scale, controlled studies are necessary to validate benefits, identify risks, and assess long-term clinical outcomes, including hospitalization and mortality rates.

#### 3.2.2 Reprieve system

The Reprieve System is an external, automated fluid-management platform. Mechanistically, it continuously monitors urine output through a urinary catheter interface and integrates the data with programmed intravenous loop diuretic delivery. Its adaptive control algorithm evaluates diuretic responsiveness on a minute-to-minute basis and dynamically adjusts diuretic infusion rates to achieve a predefined net fluid removal target. In parallel, the system can administer matched or strategically titrated isotonic fluid to modulate intravascular volume, aiming to preserve effective arterial blood volume and mitigate renal hypoperfusion. It eliminates the need for an implanted device or peritoneal access, which may lower procedural risks and enhance applicability for acute management (32). The TARGET-1 and TARGET-2 trials (NCT03897842) are small, non-randomized, single-arm, feasibility studies serving as the first-in-human experience of the Reprieve System. Nineteen heart failure patients with cardiorenal syndrome treated in these studies underwent 24 h of standard diuretic therapy followed by 24 h of diuretic therapy plus Reprieve therapy, serving as the controls (32). The use of reprieve was associated with to significantly increased diuresis and a net negative fluid balance when compared to the 24 h standard diuretic period. Additionally, there were reductions in central venous pressure, weight reduction, enhanced renal function, demonstrated by decreased creatinine and BUN/cystatin C, and stable hemodynamics, with no major device-related adverse events reported in the short term follow up (32). The study effects could be attributed to acute physiological endpoints with efficacy beyond the short term (24 h) largely unknown. Similar to the proof-of-concept studies for Alfapump device, the Reprieve device requires further large randomized clinical trials to guide meaningful therapeutic decision making and generalizability.

### 3.3 Interstitial modulators (shifters)

The microcirculation-interstitium-lymphatic (MIL) axis plays a major role in maintaining fluid homeostasis between the vascular system and the interstitial space. In normal physiology, fluid moves across capillary walls and into the interstitium, and then from there it is absorbed into the lymphatic system. In patients with cardiorenal syndrome, this fluid balance is altered due to high venous pressures. Combined with reduced lymphatic drainage, these elevated venous pressures alter the MIL axis leading to edema and ultimately hypoxia in the peripheral tissues (33). “Fluid-shifter” devices target this altered axis via different mechanisms to reduce interstitial edema.

#### 3.3.1 RenalGuard

T The device features a closed-loop weighing and infusion control mechanism that continually monitors a patient's real-time urine output and automatically administers an equivalent volume of intravenous saline, to maintain net intravascular volume while promoting high urine flow rates. Loop diuretics, primarily furosemide, are used to elicit rapid diuresis; the system's feedback algorithm promptly detects variations in urinary output through a precision scale or flow sensor and adjusts the intravenous fluid infusion correspondingly to align with urine loss on a milliliter-for-milliliter basis. The close loop between urine production and hydration minimizes hypovolemia and intravascular depletion, while promoting fast renal tubular flushing (34, 35). In a recent study evaluating 19 patients who were hospitalized for ADHF and exhibited symptoms of congestion despite being treated with standard doses of IV diuretics., the mean duration of RenalGuard therapy was 25 ± 1 h, and use of the device was associated with lower levels of serum creatinine and BUN (35). Additionally, there was a significantly lower level of IV diuretics utilized to achieve the expected diuretic response in combination with the device. This study's limitations, however, include its retrospective design, at a single center with a small sample size.

#### 3.3.2 AquaPASS

This device uses a novel approach that is different from every device discussed thus far in our review. The AquaPASS system enhances fluid and sodium loss via eccrine sweat glands. The two functional units of this device are a capsule and heating units with a controller. In essence, the capsule creates a warm temperature around the body, triggering activation of eccrine sweat glands that lead to perspiration. The perspiration is then instantly evaporated to maintain patient comfort and tolerability. A total of 6 normal subjects and 16 patients with HF underwent three treatment sessions with this device. Renal function and electrolyte levels were unchanged in the treated patients. The primary outcomes of the study showed that the device proved to be safe, but further studies are needed to demonstrate efficacy in treating symptoms of HF (36). In addition, the study excluded patients in NYHA class IV and those with eGFR < 15 mL/min/1.73 m^2^ so its applicability to patients with advanced cardiac and kidney disease is undetermined.

#### 3.3.3 WhiteSwell eLym

This device is based on the principle of thoracic duct decompression. By creating a low-pressure area around the outflow of the thoracic duct into the venous system, increased lymphatic flow is observed. In combination with IV diuretics, this model allows for the drainage of fluid from the interstitial space into the vasculature which will then be removed through diuresis. Proof of this concept was achieved in an animal model (sheep) (37).

The Decongestion of Excess Lymphatic Fluid via the Thoracic Duct in Acute Decompensated Heart Failure (DELTA-HF) trial is a multi-center, prospective, single-arm study that is investigating the safety and feasibility of the WhiteSwell eLym system (clinicaltrials.gov NCT05747196) in Europe that plans to enroll up to 50 people. The inclusion criteria for this study include primary hospital admission diagnosis of ADHF, a home diuretic dose of at least furosemide 80 mg, elevated BNP, and eGFR of at least 30 mL/min/1.73 m^2^ (38). Initial results of this trial were announced in February 2025 (39). The preliminarily released results show that decongestion was achieved in the first 21 patients that were treated. Additionally, only 9.5% of the treated patients were re-hospitalized for HF exacerbation. It was also announced that the eLym system was granted Breakthrough device status by the US FDA. As of the time of this writing, the outcomes data have not been published in a peer-reviewed journal.

### 3.4 Pullers

**“**Puller” devices offer an alternative hemodynamic strategy for CRS by selectively modulating venous pressures to reduce renal congestion. Rather than mechanically propelling blood forward, puller devices create a controlled resistance within the venous system, typically at the inferior vena cava. This leads to a reduction in upstream venous pressures, including renal venous pressure. This targeted unloading improves renal perfusion without significantly impairing systemic venous return (40).

The preCARDIA system employs intermittent superior vena cava occlusion to regulate preload and reduce central venous pressure without inducing hypotension. Clinical studies have demonstrated significant increases in urine output, reductions in natriuretic peptide levels, and stable renal function (41). Similarly, the Doraya catheter is an inferior vena cava flow regulator deployed distal to the renal veins to reduce renal afterload and enhance diuretic responsiveness. Early European studies demonstrated safety, increased diuresis, and stable creatinine levels (42). Renal vein–specific approaches, such as the Magenta Transcatheter Renal Venous Decongestion system (43, 70, 71) and JuxtaFlow (44), directly target renal venous hypertension. JuxtaFlow applies controlled negative pressure to the renal pelvis, increasing urine output and sodium excretion in early human studies, highlighting the therapeutic potential of renal venous unloading. These devices are still investigational, and there is a paucity of randomized controlled trials as most of them are still undergoing safety and feasibility testing. Most of the data are based on these initial trials as well as observational studies. Although most of these trials demonstrate reduction in CVP, PCWP and improvement in urinary output, clinical application remains limited given the small sample sizes, study designs and lack of predetermined primary renal end points.

#### 3.4.1 preCARDIA

The pathophysiologic concept behind the development of this device lies in the association of decompensated heart failure with elevated CVP, increased LV filling pressure, elevated pulmonary venous pressure, systemic venous congestion and reduction in eGFR. It is well-documented that occlusion of the IVC can lead to the rapid development of hypotension and shock. Occlusion of the SVC, however, could represent a novel approach to treatment of HF since the SVC only accounts for approximately 30% of venous return to the right ventricle. Occlusion of the SVC mechanically regulates preload and reduces CVP without leading to hypotension (41). The preCARDIA system is a catheter-mounted balloon occlusion and pump consol device based on this concept.

The VENUS-HF (Venus Heart Failure Early Feasibility Study) was a multicenter prospective single arm exploratory study of 30 patients designed to demonstrate the feasibility, safety, and potential benefit of intermittent SVC occlusion to reduce cardiac filling pressures and promote decongestion in patients with ADHF via the preCARDIA system. 1/5th of the patients received preCARDIA therapy for 12 h and the remaining patients received the therapy for 24 h. By the end of the 24 h treatments, right atrial pressure decreased by 34% and pulmonary capillary wedge pressure decreased by 27% and remained low up to 3 h after device removal. Circulating levels of brain natriuretic peptide decreased by 41%, and serum creatinine levels were unchanged after completion of preCARDIA therapy. Compared with the 24 h before treatment, total urine output increased 130% and net fluid output increased 156% during the 24 h preCARDIA treatment period. The changes in urine output and net fluid output were however not sustained after removal of the device (45). This study gives credibility to increased diuresis following preCARDIA treatment however this response was not sustained, therefore the feasibility of a sustained response and duration of therapy required to maintain this response if feasible remains undetermined.

In 2024, Yousefzai et al. published 90-day follow up outcomes for 13 patients who received preCARDIA therapy for 24 h at a single center. These patients were admitted for ADHF and had been started on at least 2.5 times their home diuretic or a total cumulative daily dose of 200 mg IV Furosemide with a total urine output less than 1,000 mL over 8 h. Echocardiographic, hemodynamic, and laboratory parameters were documented pre- and post- preCARDIA therapy. PCWP showed a significant decrease from 32 mmHg to 22 mmHg while RAP and mPAP showed a reduction that was not statistically significant. Urine output increased significantly, and serum creatinine was stable. There were no heart failure re-admissions at 90 days post hospital discharge (46) PreCARDIA therapy in conjunction with maximum diuretic therapy may be able to achieve maximal decongestion that would lead to a reduction in readmission rates for ADHF but larger studies would be required to establish this association.

#### 3.4.2 Doraya catheter

The Doraya catheter is an IVC flow regulator that is designed for transient use (less than 12 h). The catheter is inserted percutaneously into the IVC and deployed distal to the renal veins. It assists in the management of patients that are hospitalized for ADHF and reduces CVP and renal afterload, in conjunction with diuretics. It is comprised of a 12-F outer diameter, with a maximum frame diameter of 25 mm, compatible with standard 0.035-inch guidewires. Early results of the first-in-human use of this novel device in Europe were published in 2023 (42). The DORAYA trial (NCT03234647) was a non-randomized, open-label, single-arm, prospective study of 9 patients admitted for ADHF across four European institutions from 2018 to 2021. The primary outcomes were device or procedure-related serious adverse event (SAE) rate through 30 days, and secondary endpoints included diuresis, natriuresis, and serum creatinine levels. All patients were on a standard diuretic regimen with poor diuretic response. Patients with eGFR < 18 mL/min/1.73 m^2^ were excluded. Catheter insertion into the inferior vena cava required a mean of 31 ± 27 min. The entire treatment period, from device deployment to maintenance of a controlled IVC pressure gradient required 8.5 ± 1.5 h (range 7–11.5 h). The device was temporarily in place for up to 12 h prior to removal. The mean CVP and PCWP decreased during the procedure although only the drop in CVP reached statistical significance. Compared with baseline, mean diuresis increased in 7 patients. Serum creatinine levels remained stable throughout the study. The optimal duration of stay of this catheter is yet to be determined; studies with a larger sample size are needed that would include patients with advanced chronic kidney disease.

#### 3.4.3 Magenta transcatheter renal venous decongestion system (TRVD)

The Magenta TRVD system is an 18F self-expandable, axial flow pump that is deployed in the renal veins. The system was first tested in pigs with one kidney serving as the control. Baseline urine output was similar in the treated and untreated kidneys; however, during periods of increased IVC pressure, urine output fell in the untreated kidneys and remained stable in the treated kidneys. The authors later conducted the first-in-human feasibility study in 13 patients admitted for decompensated heart failure. Significant changes in urine output were not observed in the treated patients. Mean serum creatinine was 1.22 ± 0.35 mg/dL before and 1.16 ± 0.24 mg/dL after therapy, with decreases observed in 3 patients with baseline values >1.5 mg/dL. The patients reported clinical improvement prior to discharge, and this corresponded to a significant reduction in body weight (91.6 ± 16.2 at baseline vs. 80.4 ± 13.3 kg at discharge). Additionally, there was a 33% reduction in N-terminal pro-BNP (43). It is difficult to reconcile the significant weight loss achieved without a corresponding significant change in urine output; however, this is not unexpected given the small sample size.

#### 3.4.4 JuxtaFlow

The JuxtaFlow Renal Assist Device (RAD) is comprised of two soft helical tipped catheters which are placed in the renal pelvis under fluoroscopic guidance. The JuxtaFlow Renal Assist Device (RAD) is an investigational, minimally invasive system aimed at supporting kidney function through the modulation of intrarenal pressure dynamics. The device comprises bilateral ureteral catheters inserted cystoscopically into the renal pelvis, linked to an external controller that administers controlled low-level negative pressure (approximately −15 mmHg). The system aims to reduce downstream intratubular hydrostatic pressure, thereby enhancing the effective glomerular filtration gradient, increasing urine flow and natriuresis, and potentially alleviating hypoxia-related tubular injury in acute heart failure. The volume optimization incorporating negative pressure diuresis in heart failure (VOID-HF) trial was a multi-center, single-arm, first-in-human, double crossover, and prospective clinical trial examining the safety and outcomes of the JuxtaFlow device. Seven patients with diuretic resistant ADHF underwent RAD catheter placement and treatment for 24 h, six of whom completed the protocol. The major adverse event was gross hematuria reported in 3 patients, and one patient developed mild acute urinary retention that resolved. Renal ultrasound was performed on all patients at 7 days and were within normal limits. Overall, the trial showed successful deployment and utilization of the JuxtaFlow device in patients who underwent the procedure. The trial was not powered to demonstrate efficacy of the device, but there was a statistically significant increase in 24 h urine output and sodium excretion, that returned to baseline at the end of the treatment phase, highlighting its potential for use in cardiorenal syndrome (44).

#### 3.4.5 Nephronyx perfuser system

The Nephronyx Perfuser system is an experimental endovascular device positioned in the inferior vena cava just below the renal vein confluence. The passive stent-like design modifies local hemodynamics to decrease upstream renal venous pressure. The device enhances renal venous outflow and reduces venous congestion, which increases effective renal perfusion pressure while maintaining systemic venous return. The passive modulation of flow gradients is proposed to enhance glomerular filtration, improve renal blood flow, and facilitate diuresis. The perfuser distinguishes itself from active pump-based renal assist devices by utilizing intrinsic fluid dynamic principles for localized venous unloading, thereby representing a novel “puller” strategy in cardiorenal support. Initial mechanistic investigations and current clinical assessments seek to measure alterations in renal hemodynamics and confirm the therapeutic efficacy in individuals with increased central venous pressure and resistance to diuretics. Asmall, single-arm clinical trial (NCT05759806) is currently being conducted and results pending (47).

### 3.5 Pushers

In the management of cardiorenal syndrome (CRS), “pusher” devices represent a therapeutic strategy directed toward improving renal function by mechanically augmenting forward flow (40).

#### 3.5.1 Aortix percutaneous pump

This novel device is 6.5 cm long and 6 mm in diameter and is introduced percutaneously through an 18F catheter. The optimal location for device placement is in the descending aorta and superior to the renal arteries. The device contains expandable struts that help maintain the device's position in the aorta (48). The first in-human experience with the Aortix pump was in Paraguay in a group of six patients undergoing high-risk PCI. Up until the point of this study, there were only three commonly used percutaneous mechanical circulatory support (pMCS) devices being used—intraortic balloon pump, TandemHeart, and Impella. The authors recognized the shortcomings of these devices and identified a need for a device that can be used in patients that are at a very high risk of developing complications during PCI. The study authors found that during support with the device, mean urine output increased 10-fold and eGFR improved at discharge compared with baseline. No patients experienced hemodynamic compromise or adverse events (49). Given the small sample size and the lack of information on other factors that may have influenced diuresis such as the volume of the contrast load, larger sized studies with controls were recommended.

In the US, the first multi-day experience of the Aortix device was in a 54-year-old man (50). This patient had HFrEF (LVEF 17%) secondary to a non-ischemic cardiomyopathy and was admitted to the hospital for acute decompensated heart failure. At baseline, he was in NYHA class IV. The patient received six days of IV diuresis and inotropic support with minimal improvement in symptoms. Following placement of the device, net fluid loss in the patient was greater than 23L and cardiac output was over 35% of baseline. PCWP reduced from 33 to 14 mmHg, CVP dropped from 26 to 8 mm Hg, and serum creatinine improved from 3.0 to 1.3 mg/dL.Following this proof-of-concept case, 18 patients were observed after the placement of the Aortix pump in the CRS pilot study. These patients had acute decompensated heart failure, CRS, and persistent congestive symptoms (51). Pump therapy averaged 4.6 ± 1.6 days and yielded significant reductions in CVP as well as net fluid losses of 10.7 ± 6.5 L, reduction in PCWP, and reduction of serum creatinine. Additionally, patient-reported dyspnea scores improved. These findings prompted the need for additional studies. The DRAIN-HF trial is currently underway in the US (52). The clinical trial plans to enroll 268 patients with heart failure who will be randomized 1:1 to either Aortix implantation plus traditional diuretic medical therapy or medical therapy alone. Outcomes data on this trial are not yet available.

#### 3.5.2 Reitan catheter pump

This device is a percutaneous pump that is delivered via a 14F catheter into the femoral artery. Once advanced through the proximal descending aorta, it is advanced just distal to the left subclavian artery (53). The first in-human study involving this device was published in 2009 and included 10 patients who were undergoing high-risk PCI at a single center (54). High-risk was defined as echocardiographic or angiographic evidence of severe LV dysfunction, or by having a large area of myocardial involvement. The device was placed prior to PCI and was removed 1 to 6 h post-procedure in all 10 patients. Additionally, a radial arterial sheath was placed to allow for pressure measurement during PCI. The clinical events that were assessed intra-procedurally to hospital discharge included non-fatal MI, death, stroke, major bleeding, and arterial complications. In 9 out of 10 patients, the pump was successfully inserted and operated. A mean radial to femoral aortic pressure gradient of 9.8 ± 2.0 mmHg was maintained following insertion. Baseline serum creatinine fell in 7 out of 9 patients post pump removal (mean reduction 11 ± 8 μmol/L) with a resulting increase in eGFR from 66.7 ± 18.1 mL/min to 74.9 ± 23.6 mL/min, showcasing the potential role of this device in the context of cardiorenal syndrome. Following this study, twenty patients were observed over a 5.5-year period at four European cardiac centers (53). Each patient had chronic heart failure and presented with acute decompensation requiring inotropic and/or circulatory support. All patients had a LVEF < 30% and a CI < 2.1 L/min/m^2^. Patients with primary RV failure, severe peripheral arterial disease, and pulmonary hypertension with a PCWP < 15 mmHg were excluded from the study. The planned duration of the device insertion was a maximum of twenty-four hours. The primary endpoints of this study were changes in CI, serum creatinine, and eGFR. Secondary endpoints were changes in CVP, urine output, PCWP, mPAP, device-related adverse events, and 30-day mortality. 18 of 20 patients recruited underwent successful RCP implantation. The mean running time in these 18 patients was 18.3 h. Serum creatinine reduced significantly from a baseline mean of 188.4 μmol/L to 160.9 μmol/L with a corresponding improvement in eGFR from a baseline mean of 37.9 mL/min/1.73 m^2^ to 45.7 mL/min/1.73 m^2^ at 24 h. In terms of urine output, baseline was at a mean of 71.2 mL/h and increased significantly to 226.8 mL/h at 12 h. At 24 h, the mean urine output was 146.1 mL/h. This device is not currently registered for early feasibility studies in the US.

#### 3.5.3 Second heart assist pump

This device offers a unique design that is highly efficient and has the potential to eliminate complications associated with the use of other devices, particularly stroke. It is a 13.5F pump that is housed inside a stent-cage delivered percutaneously into the aorta approximately 10 cm above the renal arteries. The device operates at a mere 7,500 revolutions per minute. It reduces cardiac filling pressures and improves cardiac output by reducing afterload via activation of the impeller blades, which pull blood down through the pump, thereby generating up to an additional 2.5 L of augmented pulsatile flow over native CO to the kidneys and the rest of the body. This up to 50% increase in renal blood flow over baseline can alter the intrarenal vasoconstriction associated with low CO in HF, resulting in an increase in urine output, improvement in kidney function, and faster decongestion. There are currently two device platforms available: the Freedom Wireless Platform and the Whisper percutaneous mechanical circulatory support device. It was recently announced that a series of case studies was completed in Paraguay on 6 patients who received the Whisper device (55). Early *in vitro* data that showed that the Whisper device may be associated with lower rates of hemolysis, a common adverse complication of the currently used mechanical circulatory support devices (56). Five Whisper devices were operated at 10,000 RPM, studied against the Impella CP, which was operated at 44 133 ± 606 RPM to match the flowrate of the Whisper device. The study used the normalized and modified index of hemolysis (NIH and MIH respectively). Compared with the Impella CP, the Whisper had significantly lower NIH and MIH. Currently, there are proof of concept comparative studies or randomized clinical trials examining either Whisper or Freedom devices in the United States.

#### 3.5.4 ModulHeart

This novel device is comprised of 3 endovascular pumps that are inserted in series and assembled in parallel in the descending aorta. This design allows for higher flow than that of a single pump which is associated with less damage to blood. An adverse consequence of most transcatheter heart pumps is the destruction of von Willebrand factor. This is associated with increased risk of bleeding as well as gastrointestinal angiodysplasia, which has been observed in LVADs as well as Impella devices. The unique design of the ModulHeart device allows for an overall higher degree of flow at lower speeds, which results in lower damage to blood factors. In 2024, this design was studied using a swine model (57). The investigators found that the use of the ModulHeart resulted in preservation of VWF, compared to a 27% and 19% reduction in VWF activity with the Impella CP and 5.0 devices, respectively.

The first-in-human experience of this device was at a single-center in Paraguay (58). Four patients who required nonurgent PCI had the device inserted at the beginning of the procedure and removed following completion. The primary endpoints of the study were procedural success, and secondary endpoints included cardiac output, CVP, pulmonary artery pressure, LVEDP, periprocedural renal function, and serum creatinine level. The mean urine output at baseline 3 h before the procedure was 40 ± 5 cc/h. Urine output increased by approximately 9-fold after 15 min of support compared with baseline. Urine output remained elevated after the procedure; however, there was no significant change in the serum creatinine level or eGFR. The ModulHeart device is currently undergoing early feasibility testing in the US (Clinicaltrials.gov ID NCT06174623) (59). The inclusion criteria for this study include admission to the hospital for ADHF, age ≥ 21 years old, diuretic resistance, and clinical signs of congestion. Outcomes data for this study are not currently published.

### 3.6 Selective intrarenal drugs

Selective intrarenal drugs, as the name implies, entails infusing vasodilator medications selectively through the infrarenal artery with the aim of reducing renal vasoconstriction and consequently, improving kidney function (8). Fenoldopam is a dopamine 1 receptor agonist that reduces peripheral vascular resistance in the capillary beds of the kidneys. Although a RCT showed that IV fenoldopam did not prevent contrast associated acute kidney injury in patients with chronic kidney disease, it is presumed that this may have been due to an inability to deliver an effective renal dose of the medication (60). Delivering the medication directly to the kidneys should reduce systemic levels and blood pressure lowering effects while increasing the local concentration and GFR at the same time. The Benephit catheter was designed to allow for delivery of these medications directly to the renal arteries.

#### 3.6.1 Benephit catheter

Inserted percutaneously through the femoral artery, the Benephit is a bifurcated catheter that enables selective infusion of medications into both renal arteries. In 2006, a randomized, partial crossover, open label trial that studied 33 patients undergoing coronary angiography was published. The patients were randomized 1: 2 to either control or fenoldopam (which was initially given IV and then crossed over to IR via benephit). The patients who received IR fenoldopam had significantly higher nadir systolic BP, renal plasma flow and GFR as well as significantly lower plasma fenoldopam levels. GFR remained higher in the patients who received IR fenoldopam compared to controls two hours after medication discontinuation (61). Following this, a post market registry following patients who received targeted renal therapy (TRT) via the Benephit catheter [Benephit System Renal Infusion Therapy (Be-RITe!) multicenter registry] was developed. 501 patients undergoing peripheral or coronary intervention/angiography or cardiovascular surgery and considered to be at high risk for contrast-induced nephropathy were enrolled. Patients received either intrarenal B-type natriuretic peptide, alprostadil, sodium bicarbonate or fenoldopam via the Benephit catheter. Of the 285 patients who received fenoldopam, the incidence of CIN at 48 h was 71% lower than predicter by the Mehran risk score. The mean cannulation time was 2 min with successful bilateral renal artery cannulation in 94.2% with a conclusion that this was a safe and simple to use procedure. The trials involving IR fenoldopam have been in the context of the prevention of contrast induced kidney injury in patients undergoing PCI and it is unclear as to whether these results can be extrapolated to patients with cardiorenal syndrome and diuretic resistance (62).

## 4 Patient selection for device-based therapies in cardiorenal syndrome: identifying diuretic-refractory congestion and optimal timing of intervention

Advances in contemporary management of heart failure and cardiorenal syndrome encourage the application of precision medicine. Primary indications for escalation to device therapy is based on residual congestion, poor diuretic response, and sodium avidity rather than isolated decline in renal function (63). Persistent congestion identifies a high-risk phenotype among CRS patients despite stable serum creatinine. Current evidence suggests that one third of hospitalized HF patient will exhibit suboptimal response to diuretics manifesting as inadequate natriuresis and residual congestion despite aggressive intravenous therapy (4). These observations indicate that a select group of patients with acute CRS may benefit from escalation of interventions beyond pharmacologic therapy, including device-based options. Treatment with high doses of loop diuretics, even at aggressive doses, may not necessarily indicate refractoriness (64). In hospitalized patients, transient elevation in serum creatinine may represent hemodynamic changes and appropriate decongestion instead of intrinsic renal injury (65). Therefore, worsening renal function in isolation should not automatically prompt de-escalation of GDMT or progression to device therapy (65, 66). Rather, persistent congestion, reduced natriuresis, and diminished urine output, even with optimized dosing, more precisely indicates patients for whom pharmacologic interventions are unlikely to be effective.

The timing for device implementation has been under debate and investigation. Propositions for early implementation are based on potential advantage of disease modification and prevention of diuresis refractoriness through proactive management of hemodynamic changes associated with acute CRS (67). Alternatively, early device escalation may expose patients to unnecessary procedural risks. An individualized strategy is essential, incorporating clinical congestion, objective measures of diuretic response, and hemodynamic profiling in selecting appropriate individuals for device therapy. Future research should aim to enhance the characterization of phenotypic markers of diuretic refractoriness, establish validated thresholds for escalation to device therapy, and assess whether earlier, physiology-guided interventions can positively influence the natural progression of cardiorenal syndrome.

## 5 Proposed stepwise clinical application algorithm for device therapy implementation in CRS

**Figure 1:**
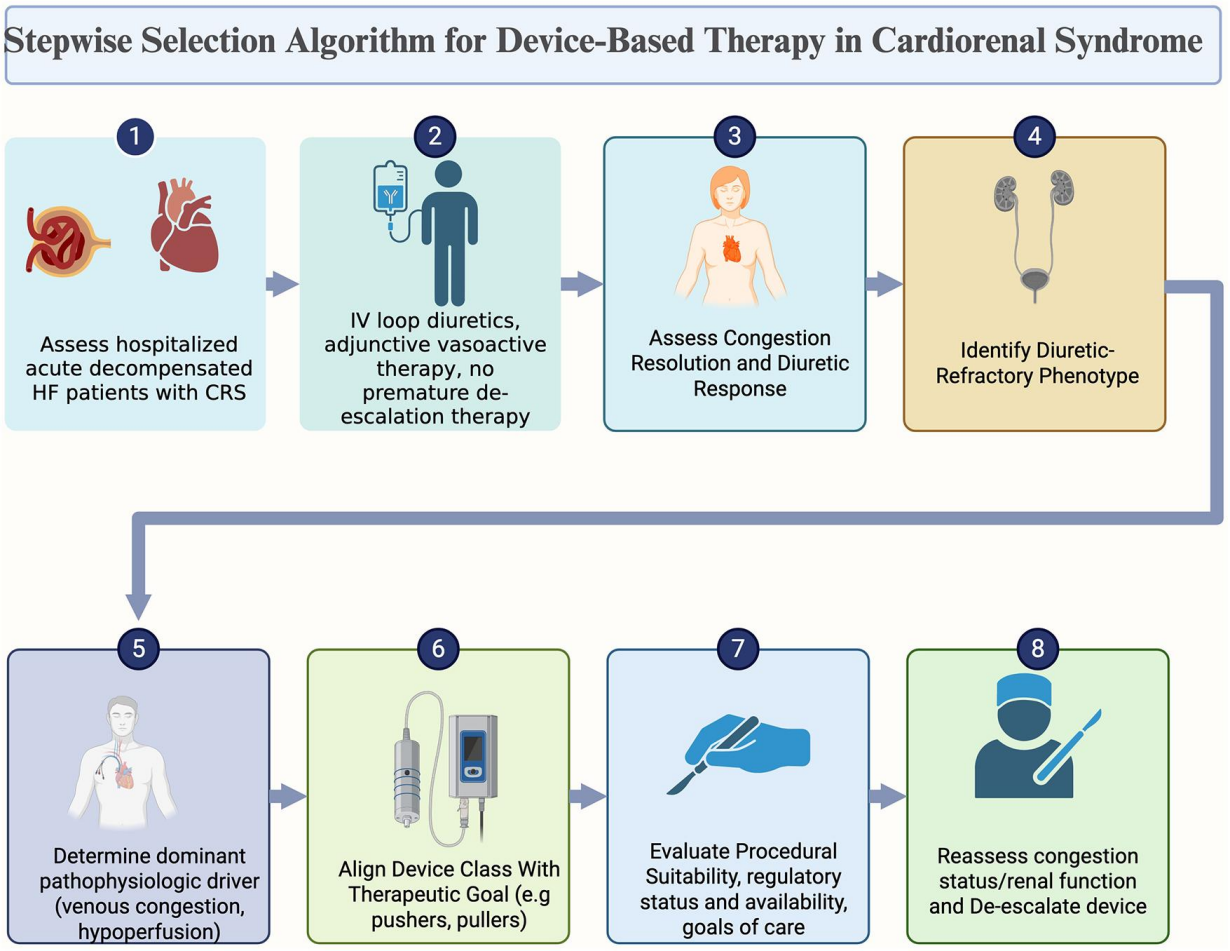
A step-by-step algorithm for choosing individualized device-based therapy for cardiorenal syndrome. In order to maximize renal and cardiac outcomes, the framework places a strong emphasis on identifying diuretic-refractory congestion, phenotypic segmentation based on primary pathophysiologic factors, and device mechanism alignment with treatment objectives.

Prior to contemplating escalation to device-based therapy, it is crucial to verify that patients received appropriate, guideline-directed medical management for acute decompensated heart failure. Appropriate patient selection for device therapy entails properly identifying hospitalized patients with clinical evidence of congestion, natriuretic peptides and renal dysfunction, confirming adequacy of guideline directed therapy if tolerated (Figure 1). Intravenous diuretic dosing should be optimized at doses aligned with the severity of congestion, alongside the judicious use of adjunctive pharmacologic agents like vasodilators when hemodynamically permit. Following initiation and optimization, response to pharmacologic therapy should be monitored for 24–48 h using objective markers of volume status such as urine output and natriuresis commensurate to administered diuretic dose (68). Where available, hemodynamic monitoring of arterial pressure, central venous pressure, and pulmonary capillary wedge pressure may be necessary to monitor appropriate response (4). In patients achieving resolution or near resolution of congestion should continue medial therapy optimization (4). In patients with refractory congestion despite appropriate diuretic dosing, worsening renal function, hemodynamic instability limiting pharmacologic therapy, the next critical step may be phenotypic and hemodynamic profiling to determine the dominant pathophysiologic driver which could aid in appropriate intervention escalation and device selection.

In patients with predominantly central venous congestion, pullers and reducer devices may be more appropriate. In those with low forward flow phenotype, pusher devices may offer the greatest physiology benefit. In the subgroup of patients with severe interstitial edema and lymphatic dysfunction phenotype, interstitial modulator treatments focused at improving lymphatic outflow or targeting tissue-level fluid buildup may be the most beneficial. Finally, for patients at high risk of over diuresis and CRS as indicated by hypotension, renal dysfunction, or intolerance to additional pharmacologic decongestion, fluid redistribution devices that manage volume without significant net fluid loss may be preferred. Thorough evaluation of procedural feasibility and risks are necessary prior to device placement. Since outcomes may be affected by procedural volume and operator expertise and institutional competence with the device technology these factors should also be considered. Furthermore, the status of regulatory approval, local availability, and reimbursement may influence feasibility. It is essential to integrate patient goals of care and preferences into the decision-making process to ensure that device-based interventions are consistent with overall treatment objectives and anticipated quality-of-life outcomes.

Device therapy de-escalation strategy should be instituted at the time of implementation and appropriate protocol proposed. Upon attainment of clinical stability and effective decongestion, therapeutic strategies should revert to optimized pharmacologic management, if feasible. Ongoing evaluation should also address the need for escalation to advanced heart failure therapies. This iterative approach emphasizes continuous reassessment and timely de-escalation, ensuring that device-based therapies are integrated thoughtfully into the longitudinal management of cardiorenal syndrome.

## 6 Challenges and considerations

To differentiate between isolated creatinine increases that do not represent clinically actionable CRS from actual cardiorenal pathology, proper phenotyping and patient selection are crucial. These could present a challenge in the effective implementation of device intervention (63). Device therapies for CRS are primarily investigational and have not been fully incorporated into established care pathways for heart failure or chronic kidney disease. Devices are presently considered supplementary options for patients refractory to guideline-directed medical therapy, typically within clinical trial environments or specialized centers that manage complex acute decompensated heart failure with concurrent deteriorating renal function (69). Operator experience and procedural learning curve are important factors for innovative CRS devices. Many circulatory support devices necessitate specialist training for insertion and hemodynamic adjustment. It is likely that outcomes could improve with greater operator familiarity and protocol refinement as clinical use expands. Finally, regulatory approval for CRS-specific devices remains limited, with most innovations currently in early-stage or feasibility trials and no broad commercial approval. Reimbursement considerations are often complicated by limited evidentiary support and the investigational nature of numerous devices. Therefore, expanded coverage will probably rely on compelling evidence showing significant clinical advantages and cost-effectiveness.

## 7 Conclusion and future direction

Current evidence supporting the devices discussed in this review lack large, randomized controlled clinical trials. However, initial clinical observations across various platforms indicate that these interventions are typically feasible and safe, demonstrating consistent short-term improvements in critical physiological parameters, such as venous congestion, cardiac filling pressures, renal perfusion indicators, and overall fluid balance.Despite these promising mechanistic indicators, significant uncertainties persist. It is unclear whether device-mediated hemodynamic and decongestive benefits last beyond the duration of active support, or whether they translate into long-term improvements in clinically meaningful outcomes such as length of stay, renal recovery, recurrent congestion, rehospitalization, or survival. Furthermore, the ideal patient selection, timing of intervention within the CRS trajectory, and length and intensity of device support remain unknown. The opportunity for synergistic deployment of complementary devices, aimed at distinct DRI2P2S pathways requires further exploration. Thus, integration of these devices into patient care would require an individualized approach until larger clinical trials can establish broad guidelines and standards of care. Despite existing gaps, the increasing mechanistic coherence, reproducible physiological signals, and alignment with current pathophysiologic models support the perspective that device-based modulation of cardiorenal interactions signifies a promising advancement in the management of acute cardiorenal syndrome.

The algorithmic framework proposed in this review offers a conceptual roadmap; however, its clinical applicability is currently constrained, as most of them are not readily commercially available and are primarily utilized in investigational trials. Future efforts must focus on expanding beyond controlled trial environments and implementing thorough post-marketing evaluations to effectively translate these mechanistic insights into practical clinical applications. This approach facilitates iterative refinement of device-guided algorithms, thereby enabling individualized, physiology-driven interventions that may transform the management paradigm for cardiorenal patients. Ongoing trials are increasingly adopting mechanism-guided approaches, which may facilitate a transition toward precision, physiology-driven care for a population that has historically faced limitations in therapeutic options.
